# Protein kinase C is involved in the neuroprotective effect of berberine against intrastriatal injection of quinolinic acid‐induced biochemical alteration in mice

**DOI:** 10.1111/jcmm.14522

**Published:** 2019-07-18

**Authors:** Peng Liu, Yinjie Li, Xiaoxiao Qi, Jia Xu, Danyang Liu, Xuefei Ji, Tianyan Chi, Han Liu, Libo Zou

**Affiliations:** ^1^ Department of Pharmacology Shenyang Pharmaceutical University Shenyang China; ^2^ Sanhome Pharmaceutical Limited Company Nanjing China; ^3^ Department of Clinical Pharmacy Shenyang Pharmaceutical University Shenyang China

**Keywords:** berberine, motor and cognitive deficits, protein kinase C, quinolinic acid

## Abstract

Protein kinase C (PKC) shows a neuronal protection effect in neurodegenerative diseases. In this study, we test whether berberine has a positive effect on the activity of PKC in quinolinic acid (QA)‐induced neuronal cell death. We used intrastriatal injections of QA mice model to test the effect of berberine on motor and cognitive deficits, and the PKC signalling pathway. Treatment with 50 mg/kg b.w of berberine for 2 weeks significantly prevented QA‐induced motor and cognitive impairment and related pathologic changes in the brain. QA inhibited the phosphorylation of PKC and its downstream molecules, GSK‐3β, ERK and CREB, enhanced the glutamate level and release of neuroinflammatory cytokines; these effects were attenuated by berberine. We used in vivo infusion of Go6983, a PKC inhibitor to disturb PKC activity in mice brain, and found that the effect of berberine to reverse motor and cognitive deficits was significantly reduced. Moreover, inhibition of PKC also blocked the anti‐excitotoxicity effect of berberine, which is induced by glutamate in PC12 cells and BV2 cells, as well as anti‐neuroinflammatory effect in LPS‐stimulated BV2 cells. Above all, berberine showed neuroprotective effect against QA‐induced acute neurotoxicity by activating PKC and its downstream molecules.

## 1 INTRODUCTION

Berberine is an isoquinoline alkaloid extracted from the roots and stem bark of many plants, such as *Coptis chinensis* and *Berberis *sp., and has been used in Chinese medicine for the treatment of bacterial diarrhoea.^1^, ^2^ Recently, studies found that berberine exerted many new uses, preventing the oxLDL and TNFα‐induced endothelial dysfunction, regulating MicroRNAs for the potential treatment of cancer and diabetes.^3^, ^4^ Berberine is a small molecule and crosses the blood‐brain barrier easily,^5^ which is an advantage to treat neurodegenerative diseases.

Huntington's disease (HD) is an inherited dominant autosomal progressive neurodegenerative disease. Behavioural characteristics include motor, learning and memory deficits. Neuropathological characteristic is the abnormal expansion of the cytosine‐adenine‐guanine repeat in the IT15 gene of chromosome 4 causing a polyglutamine stretch in the N‐terminal region of the huntingtin protein (Htt). The mutant Htt causes selective neuronal loss in the brain, particularly in the striatum and cortex.^6^ The PKC family consists of many isoforms and plays a vital role in regulating neuronal survival, proliferation, differentiation and apoptosis.^7^, ^8^ PKC mRNA levels decrease in the brain of R6/2 transgenic HD mouse model and patients with HD.^9^, ^10^ PKC inhibition causes severe neuron death in HD Q111 transgenic mice.^11^ Therefore, activating PKC pathway may be a potential method to treat HD.

In brain kynurenine pathway, tryptophan metabolizes to quinolinic acid (QA). It has been reported that the injection of QA into the striatum of rodents can induce motor, emotional and cognitive deficits in addition to significant oxidative damage and a specific loss of GABA and Ach content.^12^ The excitotoxicity of QA can cause degeneration of striatal neurons, which mimic some symptoms of patients with HD in experimental animals.

Literature has reported that berberine can decrease mutant Htt level in HEK293 cells transfected with Htt‐120Q and in transgenic N171‐82Q HD mice.^5^ However, there are no data on the actions of berberine on PKC signalling in HD animal models. Therefore, in the present research, we want to investigate the effect of berberine on motor and cognitive deficits induced by QA injection in the striatum of mice and examine the effect of berberine on PKC signalling (PKC, GSK‐3β, ERK and CREB). We also test the neuroprotective and anti‐neuroinflammatory effect of berberine in vitro and in vivo.

## 2 MATERIALS AND METHODS

### 2.1 Materials

Berberine hydrochloride (purity ≥98%) was purchased from Dalian Meilun Biotechnology Co., Ltd. and dissolved in double‐distilled water. Quinolinic acid was purchased from Sigma‐Aldrich. Go6983 (a pan‐PKC inhibitor) was purchased from Selleck Chemicals. The rabbit polyclonal antibodies against GSK3β, p‐GSK3β(S9) and p‐PKCζ(T410) were purchased from Bioworld. The rabbit polyclonal antibodies against PKCζ were purchased from Proteintech. The rabbit polyclonal antibodies against ERK1/2, p‐ERK1/2, CREB, p‐CREB and BDNF, along with the mouse polyclonal antibodies against β‐actin and secondary antibodies, were purchased from Santa Cruz.

### 2.2 Animals

Adult male KM mice (2 months old, 22‐25 g) were purchased from Liao Ning Chang Sheng Biotechnology Co., Ltd. The mice were maintained in polyacrylic cages under standard housing conditions with a 12‐hour light/dark cycle. Food and water were provided ad libitum. All of the animal studies were performed in strict accordance with the PR China legislation on the use and care of laboratory animals and the guidelines established by the Institute for Experimental Animals at Shenyang Pharmaceutical University (Permit Number: SYPU‐IACUC‐C2016‐3‐12‐252).

### 2.3 QA‐induced excitotoxicity

The mice were anesthetized with 2.5% avertin (Sigma‐Aldrich, USA) by i.p.. The head was fixed in a stereotactic apparatus. Then, the mice received unilateral intrastriatal injections of QA (85 nmol/L1 μL PBS) or PBS at the following coordinates: +0.6 mm anterior to the bregma, +2.0 mm lateral to the sagittal suture and −3.0 mm ventral. The surgical operation and the QA dose selection were based on Chiarlone A et al's report,^13^ which could induce motor dysfunction in mice.

### 2.4 PKC inhibitor administration in mice brain

The Go6983 was continuously infused into the ventricular system at a flow rate of 0.11 μL/hours by the micro‐osmotic pump (Model 1004; ALZET). The micro‐osmotic pump was connected with a infusion cannula (Brain Infusion Kit 2; ALZET) and implanted into the dorsal third ventricle (0.5 mm posterior to bregma, 3 mm below the surface of the cranium), 30 minutes after QA injection. The 60 nmol/L dose selection was based on the instructions from Selleck Chemicals (http://www.selleckchem.com/products/go-6983.html) and Bu X et al's report,^14^ which can obviously inhibit PKC activity.

### 2.5 Drug and treatment schedule

Experiment A: Mice were divided into four groups of ten animals each, control group, QA‐injected group (model group) and QA‐injected + berberine (10 or 50 mg/kg)‐treated groups.

Experiment B: Mice were divided into four groups of ten animals each, QA‐injected group, QA‐injected + berberine (50 mg/kg)‐treated group, QA‐injected + Go6983 (PKC inhibitor) group and QA‐injected + Go6983 + berberine (50 mg/kg)‐treated group.

Berberine or vehicle was orally administered by gavage immediately from the day of the QA injection. After the behavioural test, the mice were decapitated under ether anaesthesia and the brain was dissected. Four brains were stained with 2,3,5‐triphenyltetrazolium chloride (TTC) to evaluate lesion area. Cerebral cortex was used for glutamate level and neuroinflammatory cytokines level. Striatum was used for Western blotting analysis. The two berberine doses selection were based on Jiang et al's report,^5^ which showed neuroprotective effect in mice. We also tried 90 mg/kg dose, but did not show stronger effect than the 50 mg/kg dose. N = 10 mice/group in behavioural test. N = 4 mice/group in TTC staining. N = 5 mice/group in Western blotting analysis. N = 5 mice/group in estimation of glutamate and neuroinflammatory cytokines levels.

### 2.6 Measurement of bodyweight

The bodyweights of the mice were recorded after QA injection (day 1) and on the last day of the behavioural test. Percentage change in bodyweight was calculated as (Bodyweight [last day of the behavioural test‐ day1]/ day1 × 100%).

### 2.7 Rotarod test

The rotarod test was performed as described by Chiarlone A et al and van Dellen A et al,^13^, ^15^ which was performed 5‐7 and 12‐14 days after QA injection. At 2 days prior to the test, mice were exposed to a training session to acclimatize them to rotarod performance. On the day of the test, three separate trials began at the rate of 3.5 rpm with an acceleration of 20 rpm/minutes to a maximum of 35 rpm over a period of 180 seconds in a rotarod equipment. The latency to fall values were recorded, and the average time of fall was used for the comparative analyses.

### 2.8 Novel object recognition test

Novel object recognition tests were performed 15‐17 days after QA injection, as described in our previous report.^16^ Briefly, the equipment is a square box (50 cm × 50 cm × 15 cm). First day, the mice were habituated to the equipment for 10 minutes to minimize their fear of the unfamiliar environment. On the test day, two identical objects, A1 and A2, were placed at the centre of the box. The mouse was allowed to explore the objects for 5 minutes. Then, the mouse was returned to the cage. After 1‐hour rest, object A2 was replaced with a novel object B and the mouse was returned to the box to explore the objects for 5 minutes in a retention trial. After 24‐hour rest, object B was replaced with another novel object C and the mouse was returned to the box to explore the objects for 5 minutes in another retention trial. The mouse directing the object with the nose at a distance less than 2 cm was defined as exploration. The exploration time for each object was recorded. [Time spent exploring novel object/total exploration time] was defined as the preferential index (PI).

### 2.9 TTC Staining

Triphenyltetrazolium chloride staining was performed as described in our previous report.^17^ After behavioural test, the mice were killed and the brains were sectioned at the QA‐injected position and stained with 2% solution of TTC at 37°C in the dark for 20 minutes. The lesion area was stained white, and the normal area was red. TTC‐stained sections were photographed with a digital camera, and the lesion area was analysed with an image analysis software (Image‐Pro Plus 5.1).

### 2.10 Estimation of glutamate level in mice

The content of glutamate was measured from cerebral cortex tissue by using a glutamate assay kit (Nanjing Jiancheng Bioengineering Institute), according to the manufacturer's protocol.

### 2.11 Estimation of neuroinflammatory cytokines level in mice

The concentrations of TNF‐ɑ, IL‐1β and IL‐18 were measured from cerebral cortex tissue by using ELISA kits (Boster), according to the manufacturer's protocol.

### 2.12 Western blotting analysis

Western blotting was performed as described in our previous report.^16^ Briefly, 30 μg protein was electrophoresed on 8%‐12% gradient SDS‐PAGE gel and transferred to PVDF membranes (Millipore). The membranes were blocked with 5% skim milk for 2 hours at room temperature, incubated with primary antibodies: GSK3β, p‐GSK3β(S9), p‐PKCζ(T410) (1:500), PKCζ (1:800), ERK1/2, p‐ERK1/2, CREB (1:1000), p‐CREB (1:500), BDNF (1:1000), β‐actin (1:1000) at 4℃ overnight, and then incubated with secondary antibodies for 2 hours at room temperature. Protein bands were visualized using an ECL kit (Kangwei Biotechnology). The intensity was quantified using densitometry with Quantity One 4.6.2 software (Bio‐Rad).

### 2.13 Cell viability

PC12 cells were obtained from National Infrastructure of Cell Line Resource. The PC12 cells were cultured in RPMI‐1640 with 10% foetal bovine serum in a 37°C cell incubator containing 5% CO_2_. The viability of cells was tested by MTT assay. 5 × 10^4^ PC12 cells per well were planted in 96‐well plates. Different concentrations of beberine with or without Go6983 (a PKC inhibitor) were pre‐incubated with PC12 cells for 2 hours and then incubated with 8 mmol/L glutamate for 24 hours. After that, 5 mg/mL MTT was added to each well for 4 hours. The medium was removed, and 150 μL DMSO was added to each well. The absorbance was measured at 540 nm using an ELISA reader (Thermo Fisher Scientific).

BV2 cells were obtained from National Infrastructure of Cell Line Resource. The BV2 cells were cultured in high‐glucose DMED medium with 10% foetal bovine serum. 5 × 10^4^ BV2 cells per well were planted in 96‐well plates. Different concentrations of beberine with or without Go6983 (a PKC inhibitor) were pre‐incubated with BV2 cells for 2 hours and then incubated with 8 mmol/L glutamate for 24 hours, followed by cell viability analysis as described above.

### 2.14 Estimation of neuroinflammatory cytokines level in LPS‐treated BV2 cells

5 × 10^4^ BV2 cells per well were planted in 96‐well plates. Different concentrations of beberine with or without Go6983 (a PKC inhibitor) were pre‐incubated with BV2 cells for 2 hours and then incubated with 100 ng/mL LPS for 24 hours. After that, supernatants were collected from the microglia to test the TNF‐ɑ and IL‐1β levels by using ELISA kits (Boster).

### 2.15 PKC siRNA Transfections

Control siRNA/60 nmol/L PKC siRNA (M‐091560‐01‐0005, Dharmacon) was transfected into BV2 cells using Lipofectamine 2000 (Invitrogen) according to the manufacturer's protocol. After 24 hours, BV2 cells were treated with berberine (1, 2.5 and 5 μmol/L) for an additional 2 hours. Subsequently, the cells were incubated with 100 ng/mL LPS for 24 hours. After that, supernatants were collected from the microglia to test the TNF‐ɑ and IL‐1β levels by using ELISA kits (Boster).

### 2.16 BV2‐conditioned medium treatment

After treatment with LPS and berberine, the medium was collected and centrifuged at 12 000 g for 5 minutes to remove cellular debris.^18^ 5 × 10^4^ PC12 cells per well were planted in 96‐well plates. The conditioned medium was added into PC12 cells for 24 hours, followed by the cell viability analysis as described above.

### 2.17 Statistical analysis

The data were analysed using SPSS 21.0. The statistical significance was determined using one‐way ANOVA followed by Fisher's LSD multiple comparisons test with homogeneity of variance or Dunnett's T3 test with heterogeneity of variance. The Kruskal‐Wallis test was used to analyse the data, which was not normally distributed. Experimental data are represented as means ± SD or SEM. A *P* < 0.05 indicates statistical significance.

## 3 RESULTS

### 3.1 Effects of berberine on bodyweight, motor and cognitive impairments induced by intrastriatal QA injection

**Figure 1:**
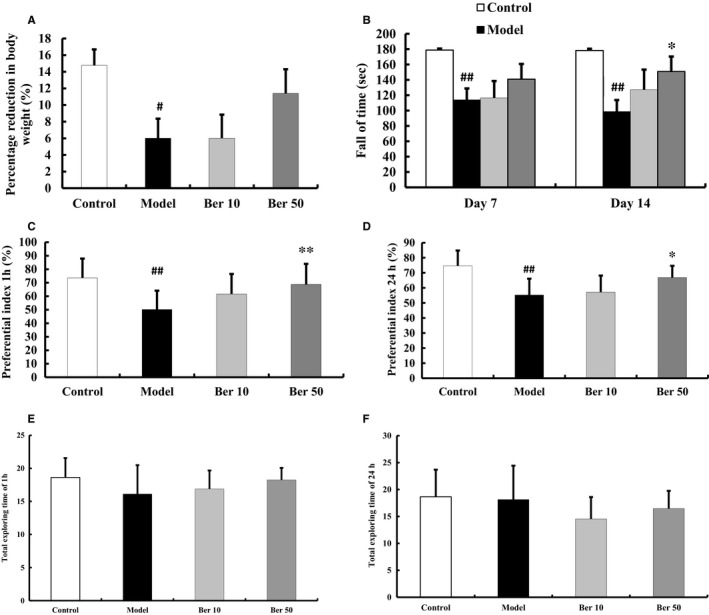
Effects of berberine on bodyweight and behavioural test in QA‐injected mice. A, QA‐injected mice exhibited a decrease in bodyweight compared with control mice. B, QA‐injected mice exhibited impairment of motor coordination in rotarod test. C‐D, QA injection impaired memory recall and visual recognition in novel object recognition test. E‐F, No significant differences in the total exploring time of 1 or 24 hours were found among the groups in novel object recognition test. All these effects were attenuated by treatment with berberine. The results of bodyweight are expressed as the mean ± SEM. The results of the behavioural test are expressed as the mean ± SD. n = 10 mice/group; ^#^ *P* < 0.05, ^##^ *P* < 0.01 vs control; *P < 0.05, **P < 0.01 vs model

QA injection reduced bodyweight on the last day of the behavioural test compared with the control group (*F*(3, 36) =2.893, *P* < 0.05, post hoc, *P* < 0.05, Figure 1A). Berberine (50 mg/kg) treatment resulted in a 5.3% increase in bodyweight compared with the QA‐treated group, though not statistically significant (*P* = 0.141, Figure 1A).

The rotarod test was performed in mice to evaluate the motor coordination after QA administration. Compared with the control group, intrastriatal QA injection shortened the latency to fall values on days 7 and 14 after QA injection (*P* < 0.01, Figure 1B). Berberine (50 mg/kg) treatment improved rotarod performance (*P* < 0.05, Figure 1B).

The novel object recognition test was performed in mice individually to evaluate the recall memory after QA administration. The PI for novel object B was decreased in the QA group (*F*(3, 36) =4.895, *P* < 0.01, post hoc, *P* < 0.01, Figure 1C), indicating that QA injection impaired memory recall and visual recognition. 50 mg/kg berberine attenuated QA injection‐induced decrease of PI (*P* < 0.01, Figure 1C). Similar results were observed with novel object C. The PI for QA group was decreased (*F*(3, 36) =8.079, *P* < 0.01, post hoc, *P* < 0.01, Figure 1D). 50 mg/kg berberine prevented these decreases (*P* < 0.05, Figure 1D). No significant differences in the total exploring time of 1 or 24 hours were found among the groups (1 hour: *F*(3, 36)=1.404, *P* = 0.257; 24 hours: *F*(3, 36)=1.491, *P* = 0.233, Figure 1E, F). Therefore, we believed that the exploration of the objects did not interfere by motor impairment. Literature reported that QA could induce motor and spatial memory impairments from 3 days to 10 weeks after intrastriatal injection.^13^, ^19^, ^20^ And treatment with berberine for 14 days significantly prevented cognitive deficits induced by Aβ or scopolamine.^21^, ^22^ Based on the above studies, we chose days 7 and 14 after QA injection to test motor impairment and days 15‐17 after QA injection to test cognitive impairment.

### 3.2 Effects of berberine on QA‐induced motor and cognitive impairments with Go 6983 infusion

**Figure 2:**
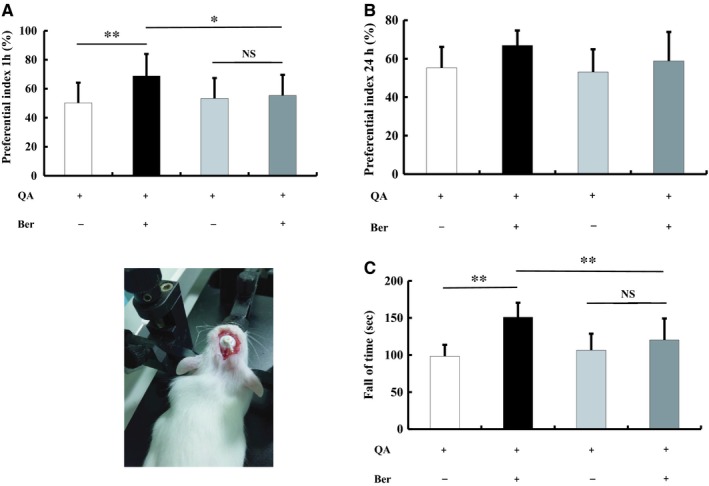
Effects of berberine on QA‐induced motor and cognitive impairments with Go6983 infusion. Go6983 infusion neither alleviated nor worsened cognitive and motor function (A‐C). But, the positive effect of berberine on mice in novel object recognition test (A) and rotarod test (C) was partially blocked by Go6983 infusion. The results of the behavioural test are expressed as the mean ± SD. n = 10 mice/group; *P < 0.05, **P < 0.01

There was no significant difference of QA group and QA + Go 6983 group in the latency to fall values and PI values (Latency to fall values: *F*(3, 36) =10.943, *P* < 0.01, post hoc, *P* = 0.411; PI for 1 hour: *F*(3, 36) =3.296, *P* < 0.05, post hoc, *P* = 0.630; PI for 24 hours: *F*(3, 36) =2.689, *P* = 0.061, Figure 2A‐C). That is to say, Go 6983 infusion neither alleviated nor worsened motor and cognitive function. However, compared with QA + berberine group, the positive effect of berberine on mice in rotarod test and novel object recognition test was partially blocked by Go 6983 infusion (Latency to fall values: *P* < 0.01; PI for 1 hour: *P* < 0.05, Figure 2A‐C).

### 3.3 Effects of berberine on QA‐induced striatal lesion area

**Figure 3:**
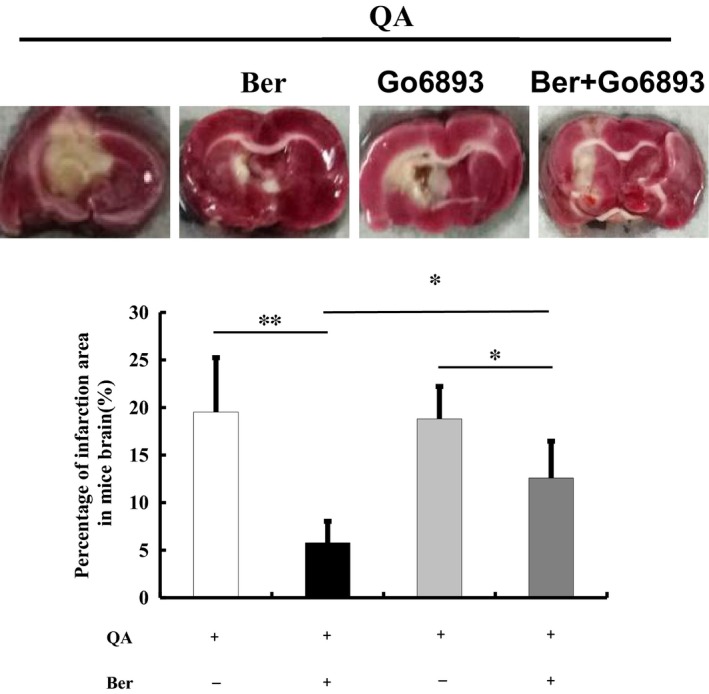
Effects of berberine on QA‐induced striatal lesion area. Brain striatal lesion size was evaluated by TTC staining. QA injection caused brain infarct injury in both the striatum and cortex. Berberine (50 mg/kg) treatment decreased lesion area percentage. Go6983 infusion partially blocked the neuroprotective effect of berberine. All of the results are expressed as the mean ± SD. n = 4/group; *P < 0.05, **P < 0.01

Brain striatal lesion size was evaluated by TTC staining at the end of the novel object recognition test. There was brain infarct injury after QA injection in both the striatum and cortex. The lesion area percentage was 19.52 ± 5.72% in QA group and 18.81 ± 3.41% in QA + Go 6983 group (*F*(3, 12) =10.177, *P* < 0.01, post hoc, *P* = 0.806, Figure 3). Berberine (50 mg/kg) treatment prevented this brain injury (*P* < 0.01). Compared with QA + berberine group, the neuroprotective effect of berberine was partially blocked by Go 6983 infusion (*P* < 0.05, Figure 3).

### 3.4 Effect of berberine on the expressions of PKC and its downstream molecules

**Figure 4:**
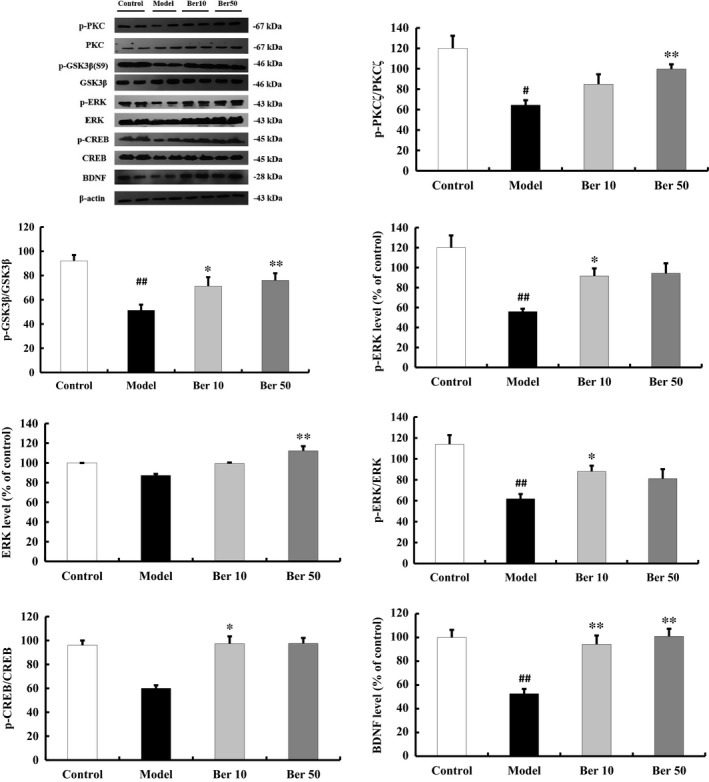
Effect of berberine on the expressions of PKC and its downstream molecules in the striatum. Berberine (50 mg/kg) increased the phosphorylation levels of PKC, GSK‐3β, ERK and CREB, and the expression level of BDNF. All of the results are expressed as the mean ± SEM. n = 5/group; ^#^ *P* < 0.05, ^##^ *P* < 0.01 vs control; *P < 0.05, **P < 0.01 vs model

PKC could regulate the activities of GSK3β and ERK.^23^ And we examined the expression levels of PKC and its downstream molecules, GSK‐3β, ERK and CREB, to investigate the role of the PKC pathway in the neuroprotective effect of berberine. QA injection decreased p‐PKC/PKC (*F*(3,16) =7.634, *P* < 0.01, post hoc, *P* < 0.01, Figure 4), p‐ERK level (*F*(3,16) =8.837, *P* < 0.01, post hoc, *P* < 0.01, Figure 4), p‐ERK/ERK (*F*(3,16) =8.952, *P* < 0.01, post hoc, *P* < 0.01, Figure 4), p‐GSK‐3β/GSK‐3β (*F*(3,16) =8.183, *P* < 0.01, post hoc, *P* < 0.01, Figure 4) and BDNF level (*F*(3,16) =13.348, *P* < 0.01, post hoc, *P* < 0.01, Figure 4). Treatment with berberine increased the p‐PKC, p‐ERK, p‐CREB, p‐GSK‐3β, and expression level of BDNF (*P* < 0.05, Figure 4).

### 3.5 Effect of berberine on the content of glutamate and proinflammatory cytokines

**Figure 5:**
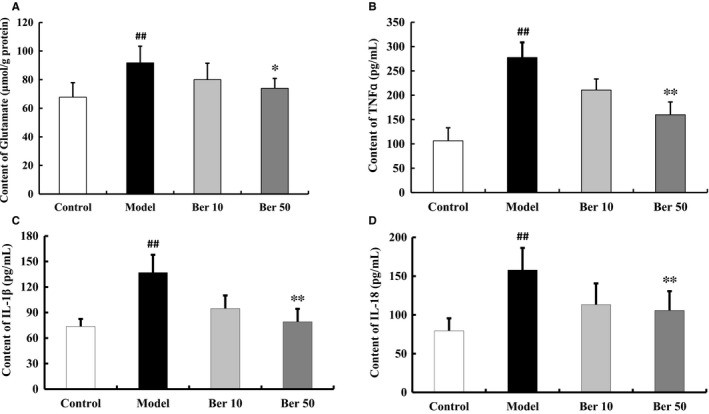
Effect of berberine on glutamate and neuroinflammatory cytokines levels in cerebral cortex. QA injection enhanced the glutamate level (A) and the content of TNF‐ɑ (B), IL‐1β (C) and IL‐18 (D). These effects were attenuated by treatment with berberine. All of the results are expressed as the mean ± SD. n = 5/group; ^##^ *P* < 0.01 vs control; *P < 0.05, **P < 0.01 vs model

Huntington's disease animal models showed an increase level of glutamate. Intrastriatal administration of QA enhanced the glutamate level in cerebral cortex (*F*(3, 16) =5.058, *P* < 0.05, post hoc, *P* < 0.01, Figure 5A). 50 mg/kg berberine decreased glutamate level in cerebral cortex (*P* < 0.05, Figure 5A).

The content of TNF‐ɑ, IL‐1β and IL‐18 was increased in the QA group (TNF‐ɑ: *F*(3, 16) =37.108, *P* < 0.01, post hoc, *P* < 0.01; IL‐1β: *F*(3, 16) =16.732, *P* < 0.01, post hoc, *P* < 0.01; IL‐18: *F*(3, 16) =8.717, *P* < 0.01, post hoc, *P* < 0.01, Figure 5B‐D). Berberine treatment prevented the release of QA‐induced proinflammatory cytokines (*P* < 0.05, Figure 5B‐D).

### 3.6 Effect of berberine on LPS‐induced proinflammatory cytokines release in BV2 cells

**Figure 6:**
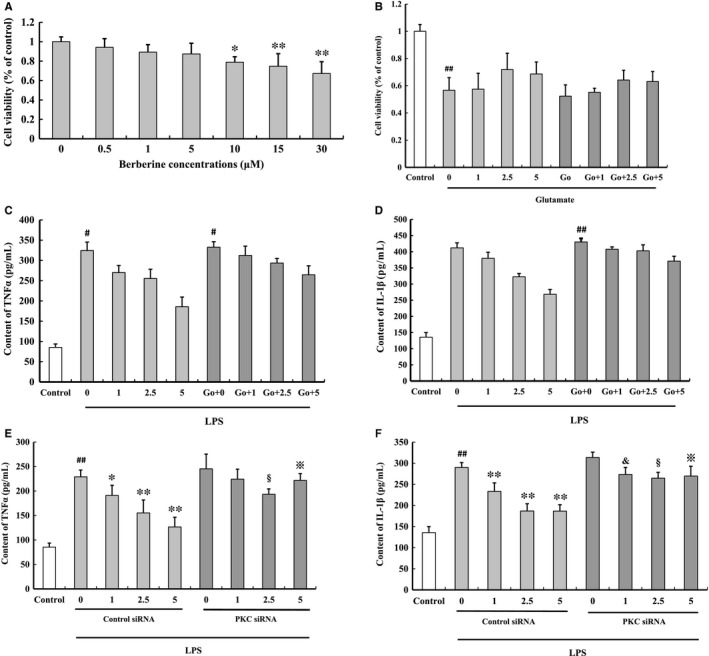
Effect of berberine on LPS‐induced proinflammatory cytokines release in BV2 cells. Berberine did not affect BV2 cell viability at concentrations from 0.5 μmol/L to 5 μmol/L (A). 2.5 μmol/L berberine slightly prevented glutamate‐induced BV2 cell death (B). Berberine decreased LPS‐induced TNF‐ɑ and IL‐1β release by microglia in a dose‐dependent manner, but Go6983 (C,D) and PKC siRNA (E,F) blocked the anti‐neuroinflammatory effect of berberine. All of the results are expressed as the mean ± SD. n = 3; ^#^ *P* < 0.05, ^##^ *P* < 0.01 vs control;*P < 0.05, **P < 0.01 vs berberine 0 μmol/L; & *P* < 0.05 vs berberine 1 μmol/L; § *P* < 0.05 vs berberine 2.5 μmol/L; ※ *P* < 0.01 vs berberine 5 μmol/L

Berberine did not affect BV2 cell viability at concentrations from 0.5 μmol/L to 5 μmol/L (Figure 6A). Therefore, we used 1, 2.5 and 5 μmol/L three concentrations to test the effect of berberine on microglia viability and the subsequent inflammatory response in glutamate or LPS‐treated BV2 cells.

BV2 cells were pre‐incubated with berberine for 2 hours and then treated with glutamate for 24 h. Berberine prevented glutamate‐induced BV2 cell death (cell viability: 1 μmol/L, 57.47 ± 11.67%; 2.5 μmol/L, 71.92 ± 11.93%; 5 μmol/L, 68.63 ± 8.83%, Figure 6B). Then, Go6983, a PKC inhibitor, partly blocked the neuroprotective effect of berberine (cell viability: 1 μmol/L, 55.19 ± 2.95%; 2.5 μmol/L, 64.18 ± 7.16%; 5 μmol/L, 63.09 ± 7.33%, Figure 6B), but with no significance.

BV2 cells were pre‐incubated with berberine for 2 hours and then added LPS. Berberine decreased TNF‐ɑ and IL‐1β level in BV2 cells. This protective effect was partly blocked by Go6983 (a PKC inhibitor), but with no significance (Figure 6C‐F). This protective effect was significantly blocked by PKC siRNA (*P* < 0.05, Figure 6C‐F). These results indicated that the anti‐neuroinflammatory effect of berberine was partly by activating PKC.

### 3.7 Effect of berberine on glutamate‐induced PC12 cells death and microglia‐mediated neurotoxicity

**Figure 7:**
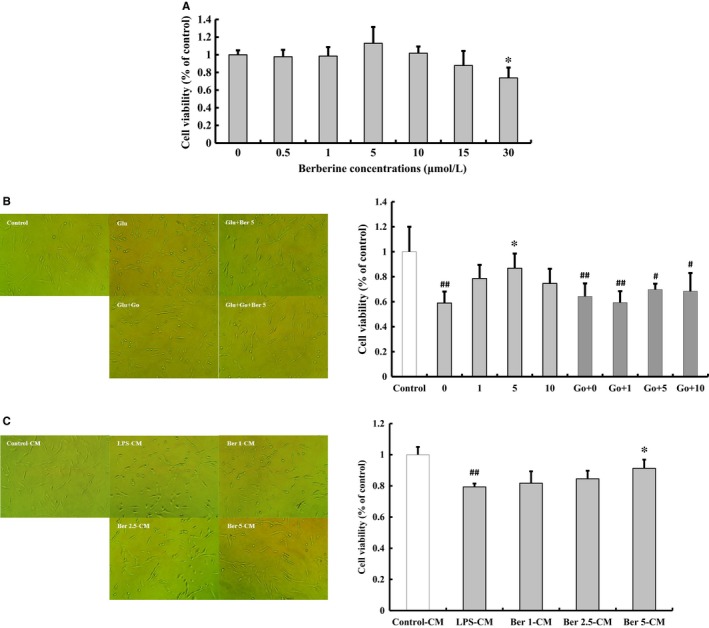
Effect of berberine on glutamate‐induced PC12 cells death and the conditioned medium (CM) from LPS/berberine‐treated BV2 cells on PC12 cells death. Berberine did not affect PC12 cell viability at concentrations from 0.5 μmol/L to 15 μmol/L (A). Berberine prevented glutamate‐induced PC12 cell death and Go6983, a PKC inhibitor, blocked the neuroprotective effect of berberine (B). CM from berberine pre‐treatment group attenuated PC12 cell death(C). Original magnification, ×20. All of the results are expressed as the mean ± SD. n = 3; ^#^ *P* < 0.05, ^##^ *P* < 0.01 vs control;*P < 0.05 vs berberine 0 μmol/L or LPS‐CM group

Berberine did not affect PC12 cell viability at concentrations from 0.5 μmol/L to 15 μmol/L (Figure 7A). Incubation with glutamate significantly decreased cell viability (58.93 ± 9.15%, *P* < 0.01, Figure 7A). Berberine significantly increased cell viability at 5 μmol/L (86.83 ± 11.71%, *P* < 0.05, Figure 7B). Go6983 blocked the neuroprotective effect of berberine (cell viability: 1 μmol/L, 59.31 ± 9.10%; 5 μmol/L, 69.77 ± 4.62%; 10 μmol/L, 68.34 ± 14.59%, Figure 7B). These results indicated that the neuroprotective effect of berberine was partly by activating PKC.

Conditioned medium (CM) from LPS‐treated BV2 cells significantly decreased the viability of PC12 cells. The CM from berberine‐treated BV2 cells could prevent PC12 cell death (cell viability: 1 μmol/L, 81.73 ± 7.62%; 2.5 μmol/L, 84.56 ± 5.17%; 5 μmol/L, 91.28 ± 5.54%, Figure 7C). Therefore, we believed that the neuroprotective effect of berberine was partly by inhibiting the activity of microglia and its subsequent neurotoxicity.

## 4 DISCUSSION

Berberine has been showed to attenuate cognitive deficits in dementia animal models and improving motor deficits in Parkinson's disease animal models.^24^, ^25^ These positive effects in cognitive and motor behaviour are regulated by many factors, such as a decrease in ROS, neuron and synapse apoptosis. Jiang et al first reported that berberine could attenuate motor impairment and prolong the survival of transgenic N171‐82Q HD mice.^5^ However, the underlying mechanisms of berberine against HD are still not fully understood.

QA, a product of tryptophan metabolism in the kynurenine pathway, can activate NMDA receptors to produce excitotoxicity and cause inflammation, oxidative stress, lipid peroxidation and mitochondrial dysfunction. The injection of QA into the striatum of rodent or primate models is frequently used to evaluate the ability of candidate compounds in minimizing the striatal lesion and preventing motor and cognitive deficits that are associated with HD pathogenesis.^26^ In this study, motor deficits were evaluated by rotarod test. QA injection caused motor incoordination, which is partially in accordance with the findings of some studies.^13^, ^27^ After berberine treatment, the motor impairments were attenuated in days 7 and 14 after QA injection. Patients with HD suffer more serious declines in memory recall function versus memory storage, which is caused by neuronal and synapse loss.^28^ In this study, we used the novel object recognition test to evaluate the effect of berberine on recall memory.^29^ The injection of QA induced persistent and significant learning and memory deficits, which were partially in accordance with findings of other studies.^30^, ^31^ The recall memory of the mice was improved by berberine treatment. Progressive bodyweight decrease is a common hallmark in patients with HD. In our study, intrastriatal QA administration resulted in a drastic decrease in bodyweight, which was attenuated by berberine. Therefore, we believe that this finding elucidates potential uses of berberine to cure HD.

PKC is important for regulating neuronal survival. Activated PKC shows a neuroprotective effect in neurodegenerative diseases.^32^ Some publications reported that inhibition of PKC isoform ζ could induce apoptosis in neurons and cancer cell lines 33 as PKC ζ, the downstream effector of PI3K, promotes cell proliferation and survival.^34^ PKC is the upstream target to regulate the activity of GSK3β and ERK.^23^ GSK3β and ERK play key roles in the regulation of some neuron survival signalling pathways. CREB is the downstream molecule of ERK. PKC activity is decreased, and the PKC activator can attenuate cognitive impairment and proinflammatory signals in APP/PS1 transgenic mice.^12^, ^35^ ɑ‐synuclein overexpression could decrease PKC and ERK activity, which further inhibits CREB transcription.^34^ PKC mediates retinoic acid induction of DARPP32 in medium‐sized spiny neurons, which is decreased in human HD. Therefore, we tested the effect of berberine on PKC and its downstream molecules. Berberine treatment could stimulate PKC/ERK/CREB, further increase the expression of BDNF and inhibit the PKC downstream effector GSK3β in QA‐injected mice. To further evaluate the role of PKC in the process of berberine reversing motor and cognitive deficits, we infuse Go6983, a pan‐PKC inhibitor by micro‐osmotic pump, to disturb PKC activity in mice brain. And we found that the positive effect of berberine on mice in rotarod test and novel object recognition test was partially blocked by Go 6983 infusion. The neuroprotective effect of berberine in TTC staining was also partially blocked by Go 6983 infusion. Therefore, the process of berberine attenuated QA‐induced motor deficits and striatal lesion was at least part through PKC pathway.

Microglia activation causes chronic neuroinflammation in neurodegenerative diseases.^36^, ^37^ Activated microglia‐released proinflammatory cytokines stimulate MAPK pathways, PKC pathways, PI3K/Akt pathways and NMDA receptors. Furthermore, the proinflammatory cytokines cause neuronal death, synaptic damage and glutamate release. Inhibiting progressive neuroinflammation is an important mechanism for protecting neuronal cells.^38^, ^39^, ^40^ Decreasing proinflammatory cytokines may be a therapeutic target in HD treatment. In this study, berberine treatment significantly attenuated QA‐increased TNFɑ, IL‐1β and IL‐18 contents in cerebral cortex. In vitro study, we found that pre‐incubated berberine showed neuroprotective effect on PC12 cells, which treated with the conditioned medium of LPS‐stimulated BV2 cells. PKC inhibitor and siRNA significantly blocked the anti‐neuroinflammatory effect of berberine in LPS‐treated BV2 cells. All the results indicated that berberine may have a potential use in HD therapy by inhibiting neuroinflammation via activating PKC.

Glutamate, the most important excitatory neurotransmitter, mediated excitotoxicity to induce brain tissue damages. NMDA receptors are hyperactive in striatal neurons of many HD animal models.^41^ In this study, QA as an NMDA receptor agonist significantly enhanced glutamate‐mediated excitotoxicity and induced serious neuronal degeneration. Berberine significantly decreased the glutamate level in cerebral cortex. Furthermore, in vitro study, we found that berberine decreased the PC12 cell and BV2 cell deaths induced by glutamate, and PKC inhibitor could block the neuroprotective effect of berberine.

**Figure 8:**
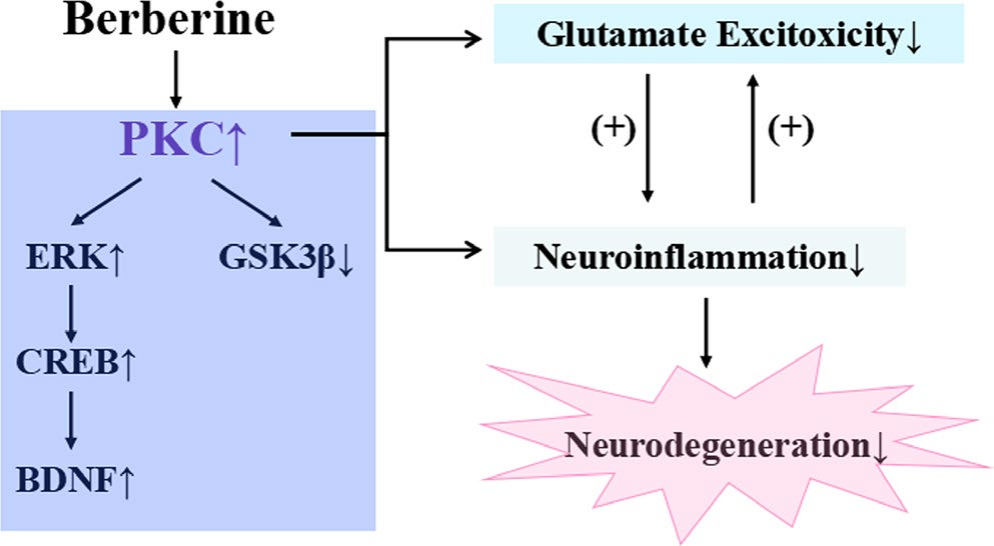
Berberine regulates PKC signalling; further, attenuates proinflammatory cytokine release; and alters glutamate levels

In conclusion, berberine is protective against intrastriatal injection of QA‐induced motor and cognitive impairments. Moreover, berberine regulates PKC and its downstream molecules, GSK‐3β, ERK and CREB; increases BDNF levels; further, attenuates proinflammatory cytokine release; and alters glutamate levels (Figure 8). Taken together, berberine showed neuroprotective effect against QA‐induced acute neurotoxicity by activating PKC and its downstream molecules. However, it remains unclear how berberine activates PKC, and whether berberine can directly be associated with PKC to regulate its function. These questions require more in‐depth studies to clarify.

## CONFLICT OF INTEREST

The authors confirm that there are no conflicts of interest.

## AUTHOR CONTRIBUTION

P. Liu conceived the experiments, contributed to research data and drafted the manuscript; Y. Li, X. Qi and D. Liu contributed to research data; H. Liu and J. Xu participated in the model design and analysis; X. Ji and T. Chi revised the manuscript. L. Zou revised the manuscript and supervised the analysis.

## Data Availability

The data that support the findings of this study are available from the corresponding author upon reasonable request.
